# Examining Associations Among Orthorexia Nervosa and Anthropometric Factors and Lifestyle Habits in an Italian University Community

**DOI:** 10.3390/nu17030537

**Published:** 2025-01-31

**Authors:** Giuseppina Augimeri, Martina Marchese, Pierluigi Plastina, Daniela Bonofiglio

**Affiliations:** 1 Department of Pharmacy, Health and Nutritional Sciences, University of Calabria, 87036 Arcavacata di Rende, CS, Italy; giuseppina.augimeri@unical.it (G.A.); martinamarchesee@libero.it (M.M.); pierluigi.plastina@unical.it (P.P.); 2 Centro Sanitario, University of Calabria, 87036 Arcavacata Di Rende, CS, Italy

**Keywords:** orthorexia nervosa, ORTO-15, Mediterranean diet adherence screener, Mediterranean lifestyle index

## Abstract

Background/Objectives: Orthorexia nervosa (ON) is characterized by an obsession with rigid dietary rules, which leads to an emphasis being placed on food purity and health. Exploring the prevalence rates and understanding the potential risk factors associated with ON is essential for developing effective prevention and intervention strategies. This study investigated the prevalence of ON and examined different variables in associations to enhance our knowledge of their impact on ON tendency. Methods: A sample of 500 participants, including 357 women and 143 men, aged between 20 and 60, from an Italian university community was recruited to complete an online survey assessing ON, using the 15-item self-report measure ORTO-15, and Mediterranean diet adherence and lifestyle habits using the Mediterranean Diet Adherence Screener (MEDAS) and the Mediterranean Lifestyle Index (MEDLIFE) questionnaires, respectively. Student’s *t*-test, ANOVA, chi-squared test, and multiple linear regressions were used for analyses. Results: We found that MEDAS and MEDLIFE scores were statistically higher in males than in females, while the ORTO-15 score was significantly lower in females than in males. In the total, ON prevalence was 19.8% (women = 16.08% and men = 21.28%). Multiple regression analyses on the ORTO-15 score and different variables showed that in our population sample, ON was associated with female sex (β = −2.98; *p* = 4 × 10^−6^) and the body mass index (BMI) (β = −0.41; *p* = 6.71 × 10^−7^). When adjusting for sex and the BMI, the resulting ORTO-15 score was associated with health science faculty attendance (β = 1.26, *p* = 0.003), following a food plan (β = −3.14; *p* = 1 × 10^−7^) and carrying out physical activity (β = −1.20; *p* = 0.03). Conclusions: This study identified the importance of several factors for ON focusing on lifestyle habits that clinicians should consider when assessing patients at risk for eating disorders. Further studies are warranted to better define the diagnostic criteria of ON and develop effective prevention and intervention strategies to promote a healthy relationship with food.

## 1. Introduction

Orthorexia nervosa (ON) is a disordered and emerging eating behavior, characterized by a pathological obsession with healthy food. The term ON was first coined by Bratman and Knight in 1997 [1] to describe subjects following restrictive diets, ritualized eating patterns, and a rigid avoidance of foods deemed unhealthy or impure, driven by the desire to maximize their physical health. People with an ON tendency do not eat foods considered impure because of the presence of pesticides or artificial substances, resulting in the restricted intake of food groups such as meat, dairy, grain and cooked food [2]. In addition, orthorexic people pay extreme attention to food sourcing and processing in order to avoid cooking methods that reduce the nutritional content, and they scrutinize food packaging for potential carcinogenic compounds. As a consequence, people with an ON tendency avoid eating with other people and spend a lot of time planning meals and preparing “pure foods”, experiencing frustration and constant worry when food purity is compromised. This obsession with food purity is often associated with significant physical impairment since the attempt to achieve optimal health through attention to diet leads to malnutrition [3]. Moreover, ON is linked to psychological symptoms, including depression and anxiety, which might impact social activities, determining the loss of relationships and poor quality of life [4]. Several psychological and socio-cultural factors have been associated with ON tendency. Personality traits, such as perfectionism and high levels of neuroticism, as well as the history of diet and an unsatisfactory body perception increase the risk for developing ON [5]. Moreover, the media representation of unrealistic body standards, the persuasive influence of social media, and social pressures to conform to particular body ideals contribute to the adoption of extremely rigid eating patterns that encourage the stigmatization of certain foods [5]. To date, since ON has not been officially recognized as a psychopathological disorder in the Diagnostic and Statistical Manual of Mental Disorders (DSM-5), its diagnostic criteria have not been established, making the assessment of ON prevalence and its incidence rates difficult [6]. However, it has been observed that ON tendency increases the risk for eating disorders [7,8]. To date, ON tendency is evaluated by questionnaires [9], among which the ORTO-15 test is one of the most widely used. It aims to evaluate cognitive factors related to eating behaviors and clinical and emotional features [10]. Lower life satisfaction, higher stress, greater depressive symptoms, and lower global functioning have been described as typical clinical features of ON, which are shared with other psychopathological disorders, including the eating disorders [11]. On the other hand, ON individuals lose weight as consequence of an obsessive preoccupation with the quality of food; in contrast, individuals with eating disorders are focused on the quantity of food consumed, with weight loss serving as primary endpoint of the restricted diet [12,13]. Other common symptoms have been found between ON and obsessive compulsive disorders, including the time spent in choosing and preparing healthy food and the distress and anxiety caused by intrusive thoughts related to food, which result in wasting time and a limited social life [3]. Some authors have described that the appearance orientation and overweight preoccupation represent the main predictors of ON [14,15], suggesting that the fear of gaining weight and concerns about one’s physical appearance are the driving force for adhering to a restricted diet. In contrast, adopting a dietary pattern potentially leading to health benefits has been linked to several physical and psychological advantages. For instance, high adherence to the Mediterranean diet (MD), characterized by the high consumption of plant-based foods, moderate consumption of poultry and dairy products and low intake of red meat, is associated with a reduction in several chronic diseases, including cardiovascular, metabolic and neurodegenerative disorders [16,17,18], along with a reduced risk of depression [19], anorexia, bulimia nervosa [20] and binge eating disorder [21]. To the best of our knowledge, only one study has demonstrated that a higher adherence to the MD is associated with a higher risk for developing ON in professional athletes [22], suggesting that further research is warranted for developing effective prevention and intervention strategies to promote a healthy relationship with food.

The aim of this observational study was to evaluate the prevalence of ON and the adherence to the MD within a university community in Southern Italy in order to examine the impact of different anthropologic, sociodemographic and lifestyle factors in ON tendency.

## 2. Materials and Methods

### 2.1. Participants

Participants were recruited from the University of Calabria via an invitation to fill out a web survey sent to their institutional email and via social media platforms. In particular, the purpose of the study to the participants was explained and they were asked to give informed consent. Participants completed the questionnaire directly connected to the Google platform, which was used to create our online survey. The inclusion criteria required participants to be healthy individuals over the age of 18, recruited in the community of the University of Calabria. The self-administered structured questionnaire consisted of 68 questions divided into three parts to assess the food and lifestyle habits of the study population. This study was approved by Italian Ethics Committee of the University of Calabria, Italy (#0475765/2024, 20 December 2024).

### 2.2. Anthropologic and Sociodemographic and Lifestyle Data

The first part of the questionnaire focused on the general characteristics of the participants, including nationality, age, gender, height and weight, by which their body mass index (BMI, kg/m^2^) was calculated. In addition, the following socio-demographic information was collected: residential distribution within the University of Calabria campus, the degree program attended (health sciences, engineering and technological sciences, economic and legal sciences, social and political sciences, or humanities) and the adherence to a dietary pattern (a hypocaloric diet, the MD, a ketogenic diet, or high-protein or high-calorie diets). Moreover, the lunch location was also assessed (home, university canteen, or bar).

### 2.3. Assessment of ON Tendency

The second part of the questionnaire consisted of an assessment of ON tendency using the ORTO-15 questionnaire and the Eating Habits Questionnaire-21 (EHQ-21). The ORTO-15 questionnaire is a validated tool that measures the risk of ON based on 15 questions with Likert-scale response categories (always, often, sometimes, or never) scored from 1 to 4, with lower points indicating ON tendency [10]. According to Ramacciotti et al. [23], a cut-off at <35 indicated a tendency toward ON. The EHQ-21 is a twenty-one-item self-report questionnaire that evaluates issues related to healthy eating, knowledge of healthy eating and positive feelings about healthy eating using a four-point scale ranging from 1 (false, not at all true) to 4 (very true). Higher points indicate ON tendency [24].

### 2.4. An Assessment of the Adherence to the Mediterranean Diet Pattern

The third part of the questionnaire was composed of a modified version of the Mediterranean Diet Adherence Screener (MEDAS), including 13 items, and the Mediterranean Lifestyle Index (MEDLIFE), used as validated tools to investigate the adherence to the Mediterranean diet pattern [25,26]. Briefly, the modified version of the MEDAS questionnaire consisted of 2 questions on food consumption habits and 11 questions on food consumption frequency. MEDLIFE is composed of 6 questions querying healthy lifestyle habits such as engagement in physical activity, sleeping, and social and conviviality habits. Each question was scored 0 or 1, with higher scores indicating higher adherence to the MD.

### 2.5. Statistical Analysis

Data collected from the questionnaires were entered and analyzed using IBM SPSS Statistics version 21.0 for Windows. Data were reported as the mean and SD and statistical differences between samples were evaluated using Student’s *t*-test and ANOVA where applicable. Qualitative variables were reported as frequencies (%) and statistical differences were evaluated by chi-squared tests. The association between ORTO-15 and EQH-21 scores was evaluated using Pearson’s linear correlation index in the GraphPad-Prism 7 v.7 software program. Multiple linear regression models were fitted to analyze the association among the ORTO-15 score and food and lifestyle variables. The sample size calculation was performed using a 95% confidence interval (CI) and a margin of error (d) of 5%. A minimum number of 385 participants was requested. P-values lower than 0.05 were considered statistically significant.

## 3. Results

### 3.1. Sample Characteristics

The main characteristics of the total sample population categorized by gender are reported in Table 1. A total of 500 participants, including 143 men and 357 women, were recruited at the University of Calabria. The mean age and BMI were 23.48 ± 5.08 years old and 22.91 ± 3.95 kg/m^2^, respectively. In total, 57.8% participants lived off campus, 16.8% lived on campus and 25.4% were commuter students. The majority of participants (67.6%) attended health science courses, whereas the remaining percentage studied non-health-related programs, including engineering and technological sciences (7.4%), economic and legal sciences (3.6%), social and political sciences (5%) and humanities courses (9%). Half of the total sample had lunch both at home and in the university’s canteen, while 38.4% and 10% had lunch only at home and in the university’s canteen, respectively.

### 3.2. ORTO-15, MEDAS and MEDLIFE Questionnaires Scores in the Population

**Figure 1:**
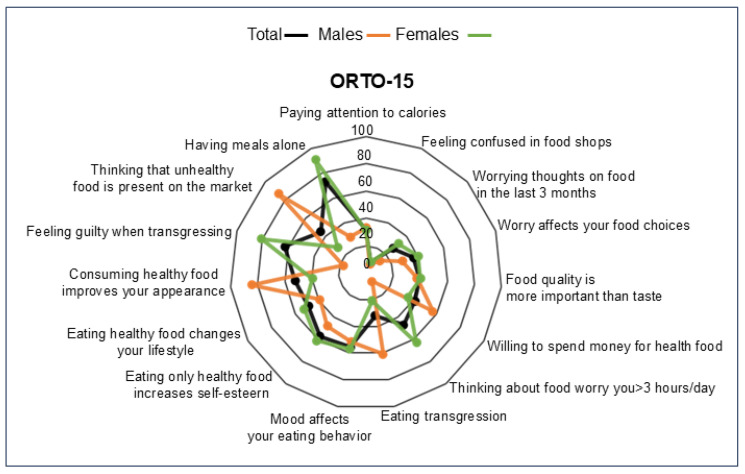
Compliance with items from the ORTO-15 questionnaire in the total sample, males and females. The radar chart displays the values of each item of the ORTO-15 score along individual axes, with the center representing 0% compliance and the outer ring indicating 100% compliance.

The ORTO-15 score was 39.63 ± 6.35 in the total population. Upon categorizing the population by gender, the ORTO-15 score was 41.06 ± 5.58 in men and 39.06 ± 6.56 in women (*p* = 0.001), suggesting that the tendency toward ON was predominantly observed in the female population (Figure 1). The EHQ-21 score was 36.52 ± 9.27 in the total population, whereas no differences were found when categorizing the population by gender (men: 37.36 ± 9.83; women: 36.23 ± 9.03, *p* = 0.26). The ORTO-15 score was positively correlated with the EHQ-21 scores (Supplementary Figure S1), further confirming the validity of the two questionnaires in assessing ON tendency.

**Figure 2:**
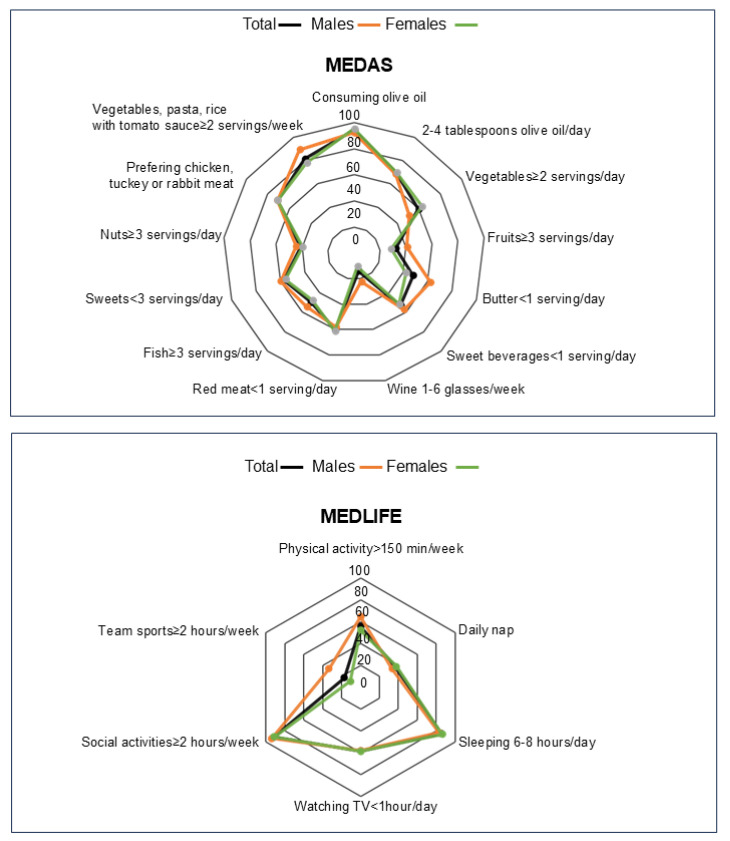
Compliance with items from the MEDAS and MEDLIFE questionnaires in the total sample, males and females. The radar chart displays the values of each item of the MEDAS and MEDLIFE scores along individual axes, with the center representing 0% compliance and the outer ring indicating 100% compliance.

In order to investigate the adherence to the Mediterranean diet pattern, we administer the MEDAS and the MEDLIFE questionnaires to our sample population, the results of which are depicted in Figure 2.

The MEDAS and MEDLIFE scores were 7.32 ± 2.24 and 3.45 ± 1.17 for the total population, respectively, indicating a moderate adherence to the Mediterranean diet pattern. Moreover, significantly higher MEDAS and MEDLIFE scores were found in men than in women (MEDAS: 7.72 ± 2.47 vs. 7.15 ± 2.12; MEDLIFE: 3.66 ± 1.24 vs. 3.36 ± 1.13) (Table 2).

### 3.3. Orthorexia Nervosa Tendency in Our Sample Population

ON tendency was observed among 19.8% of the total sample when fixing the cut-off at the 35-point threshold. ON tendencies were 16.08 and 21.28 for males and females, respectively, with no significant differences in ON tendency among gender groups at the 35- and 40-point threshold cut-off (Table 3).

### 3.4. Influence of Anthropometric Parameters and LifeStyle Habits on the Tendency Toward Orthorexia Nervosa

It has been established that ON is influenced by several factors, including demographics and psychological and sociocultural variables [5]. We observed significantly different ORTO-15 scores when categorizing the population according to the BMI (*p* = 0.003), with obese participants having a lower ORTO-15 score (36.72 ± 8.18). In addition, we found a higher ORTO-15 in participants attending health sciences courses than the other ones (40.07 ± 6.28 vs. 38.7 ± 6.44; *p* = 0.03) (Table 4). Among our sample population, 41. 2% of participants declared that they follow a specific diet; more precisely, 4% follow the ketogenic diet, 22.8% the hypocaloric diet, 4.8% the Mediterranean diet, 2.8% the high-protein diet, and 5.4% the high-calorie diet. Interestingly, a significantly different ORTO-15 score was found in the population categorized based on their food plan (*p* = 0.002), with participants following the ketogenic diet having the lowest ORTO-15 score (33.25 ± 6.965), which fell within the pathological range for ON. In addition, participants declaring to follow the hypocaloric diet, the Mediterranean diet and the high-calorie diet showed an ORTO-15 score lower than 35 (hypocaloric diet: 36.6 ± 6.12; Mediterranean diet: 36.08 ± 5.65; and high-calorie diet: 38.7 ± 5.45). In order to investigate whether food choices or lifestyle habits might influence the tendency toward ON, we examined the ORTO-15 score in relation to the participants’ answers to each item from the MEDAS and the MEDLIFE questionnaires. We observed a significantly lower ORTO-15 score in participants declaring to have more than two servings of vegetables per day from the MEDAS questionnaire (39.04 ± 6.28 vs. 40.52 ± 6.38, *p* = 0.01). Similarly, participants who engaged in more than 150 min of physical activities per week, according to the MEDLIFE questionnaire, had a significantly lower ORTO-15 score (39 ± 5.99 vs. 40.44 ± 6.71, *p* = 0.01) (Table 4).

### 3.5. Association Between ON Tendency and Different Parameters

The results of the multiple linear regression analysis are shown in Table 5. The ORTO-15 score was negatively associated with sex (*p* = 4 × 10^−6^) and the BMI (*p* = 6.71 × 10^−7^) (Model 1). When adjusting the data for gender and the BMI, the ORTO-15 score was positively associated with attendance to a health science faculty and negatively associated with both the adherence to a food plan and engagement in physical activities (Table 5).

## 4. Discussion

This study was the first to assess the prevalence of ON in an Italian university community of Southern Italy, examining different variables in association to evaluate their impact on ON tendency. In the last few years, the number of reported cases of ON has been increasingly recognized [27], even if a standardized definition of this eating disorder lacks, making it hard to compare studies, evaluate prevalence rates and identify risk factors [28]. Indeed, to date, ON is not listed by the DSM-5 [29] nor by the International Classification of Diseases (ICD-10) [30], as it is not considered an official mental disorder. Specific questionnaires and diagnostic tools have been designed to aid in the identification and evaluation of ON. Among the widely used assessment instruments, the 15-item self-report measure ORTO-15 was developed for ON based on the “Bratman Orthorexia Test” for use in an Italian population [10,23,31]. In our population sample, ON prevalence using ORTO-15 was 19.8%, affecting 21.28% of men and 16.08% of women, with a 35-point threshold. Our findings are in agreement with the data previously published [12,23]. Even the prevalence estimates for ON measured by ORTO-15 varies widely, with some studies reporting rates as low as 6.9% [32] and some as high as 90.6% [33] among specific populations. In contrast to our data, some authors reported that ON is more prevalent in men than in women [34,35], while some controversial data have been reported in the literature [36,37,38,39]. In addition, we found a lower ORTO-15 score in the female population than in the male population, which could be associated with higher levels of a positive body image among men than among women [5,40,41]. Similar inconsistencies in the literature pertain to the associations of ON with age, the BMI, and the level of education [23,42,43,44,45,46,47,48,49]. From multiple regression analysis, we found that the ORTO-15 score was associated with gender and the BMI, indicating the inverse correlation of ON tendency in men and with the BMI. These findings led us to adjust the data for sex and the BMI, whereby ON tendency was found to be positively associated with attendance to a health science faculty and negatively associated with both adhering to a food plan and physical activities.

While an obsession with pure food is pathological, adopting a healthy diet without an excessive preoccupation with healthy food consumption and punishment when violating personal rules can provide a sense of identity, offering a way to define themselves within a health-conscious community. In this context, ongoing research aims to define the motivation behind food choices that differentiate “healthy” orthorexia from ON. Depa et al. have demonstrated that weight control and health content are the main reasons that guide food choices in ON and healthy orthorexia cases, respectively [15]. The positive impact of adhering to a healthy dietary pattern, such as the Mediterranean diet, has been well established in numerous studies. It is well known that the Mediterranean diet represents one of the healthiest dietary models worldwide. The Mediterranean diet is characterized by high intake of plant-based foods, such as fruits and vegetables, a moderate intake of dairy products, fish and poultry and a low intake of red meat. In addition, regular physical activity, adequate rest and socialization during meals are also recommendations of the Mediterranean diet lifestyle [50]. Several studies have demonstrated that the Mediterranean diet reduces the risk of several chronic diseases, including cardiovascular diseases, metabolic diseases and different types of cancer [18,51,52,53,54,55,56,57,58,59]. Despite these benefits, a decrease in the adherence to the Mediterranean diet, with a shift toward a westernized diet, has been observed in recent decades [60]. According to our previous studies assessing adherence to the Mediterranean diet pattern in a country in the Mediterranean area [60,61], we found an average level of adherence to the Mediterranean diet pattern. However, we did not find any correlations between the ORTO-15 score and MEDAS and MEDLIFE. In contrast, individuals declaring to follow a dietary pattern, included the MD, showed a significantly lower ORTO-15 score compared to those who did not declare to adhere to a specific dietary pattern. Our data are in line with the findings of other authors. For instance, Rozmiarek et al. investigated ON tendency in students from health- and non-health-related fields, and they observed a higher ON tendency in subjects aware of the importance of following a health dietary pattern, suggesting that ON tendency is higher in individuals concerned with health and a proper body shape [62]. These data highlight the need to aware the population about the importance of following health dietary habits without a pathological obsession with healthy eating, which may lead to ON tendencies. ON was also positively associated with difficulties in emotion regulation and attachment-related anxiety and avoidance [63], whereas health-conscious eating behaviors have been linked to mindfulness practices [64], suggesting the importance of psychological drivers in developing ON tendency. In this context, intuitive eating and indices of positive body image have been found to be important parameters to distinguish between healthy orthorexia and a pathologically obsessive eating style such as ON [65]. Our data also showed that ON tendency was significantly lower in subjects declaring to be engaged in physical activity, which is a universal predictor of good physical and mental health. Other studies have investigated the association between physical activity and ON tendency, demonstrating that athletes have ON tendency [8,66].

Some limitations should be considered in our study. First, our study was carried out in a single center represented by an Italian University, making it difficult to draw general conclusions. Indeed, our primary objective was to generate robust findings in a cohort of an academic community, which we intend to use as a foundation for future multi-center studies. Furthermore, the population sample was composed of a higher percentage of men compared to women, resulting in an unequal distribution of participants regarding sex. Although this issue represents a limitation in our study, it reflects a broader trend in greater female involvement in nutritional studies and health interventions. Finally, additional questionnaires to estimate ON tendency should be used to strengthen the results of our study and to evaluate ON tendency more accurately.

## 5. Conclusions

This study was the first to assess the prevalence of ON in an Italian university community of Southern Italy and to identify the importance of several factors for ON focusing on lifestyle habits that clinicians should consider when assessing patients at risk for eating disorders. Our findings suggest that a lower adherence to the MD along with a rise in ON tendency could lead to public health issues, including malnutrition, poor mental health, and social isolation. Thus, further studies are needed in order to better define the clinical feature of ON and identify the predictors for its development. In addition, there is a pressing need to promote awareness about health dietary habits, emphasizing the distinction between maintaining a balanced diet, such as the MD, and developing an obsessive preoccupation with health food plans. This strategy might decrease the risks associated with ON while fostering a healthier relationship with food [60].

## Figures and Tables

**Table 1:** Characteristics of the study population.

Characteristics	Total Sample	Men	Women
Number of participants (%)	500	143 (28.6)	357 (71.4)
Age (years) mean ± SD	23.48 ± 5.08	23.10 ± 4.52	23.64 ± 5.29
BMI (kg/m^2^) mean ± SD	22.91 ± 3.95	24.37 ± 3.88	22.14 ± 3.80
Nationality, n (%)			
Italian	492 (98.4)	140 (97.9)	352 (98.6)
International	8 (1.6)	3 (2.10)	5 (1.4)
Residential distribution, n (%)			
Living on-campus	84 (16.8)	23 (16.08)	61 (17.09)
Living off-campus	289 (57.8)	86 (60.14)	203 (56.86)
Commuter students	127 (25.4)	34 (23.78)	93 (26.05)
Faculty, n (%)			
Health sciences	338 (67.6)	91 (63.64)	247 (69.19)
Engineering and technological sciences	37 (7.4)	23 (16.08)	14 (3.92)
Economic and legal sciences	18 (3.6)	7 (4.90)	11 (3.08)
Social and political sciences	25 (5)	7 (4.90)	18 (5.04)
Natural sciences	37 (7.4)	8 (5.59)	29 (8.13)
Humanities	45 (9)	7 (4.90)	38 (10.64)
Lunch location, n (%)			
Home	192 (38.4)	35 (24.47)	157 (43.98)
University canteen	50 (10)	19 (13.29)	31 (8.68)
Home and university canteen	252 (50.4)	88 (61.54)	164 (45.94)
Bar	3 (0.60)	1 (0.70)	2 (0.56)
Skipped lunch	3 (0.60)	0	3 (0.84)

BMI, body mass index; n, number; SD: standard deviation.

**Table 2:** ORTO-15, MEDAS and MEDLIFE scores in the total population and categorized by gender.

ORTO-15 Score	Total Sample, n (%)	Men, n (%)	Women, n (%)	*p*-Value
<35	99 (19.8)	23 (16.08)	76 (21.28)	0.09
>35, <40	112 (24.4)	25 (17.48)	97 (27.17)	
>40	279 (55.8)	95 (66.43)	184 (51.54)	

**Table 3:** Orthorexia nervosa tendency in the total population and categorized by gender.

Scores	Total Sample	Men	Women	*p*-Value
ORTO-15 Score Mean ± SD	39.63 ± 6.35	41.06 ± 5.58	39.06 ± 6.56	0.001
MEDAS Mean ± SD	7.32 ± 2.24	7.72 ± 2.47	7.15 ± 2.12	0.009
MEDLIFE Mean ± SD	3.45 ± 1.17	3.66 ± 1.24	3.36 ± 1.13	0.01

**Table 4:** ORTO-15 score in the sample population according to anthropometric parameters and eating habits.

Characteristics	n	%	Minimum–Maximum	ORTO-15 Score	*p*-Value
BMI					
Underweight	40	8	30–53	42.3 ± 5.53	0.003
Normal weight	354	70.8	20–53	39.71 ± 6.22	
Overweight	77	15.4	22–50	39.01 ± 6.16	
Obesity	29	5.8	24–60	36.72 ± 8.18	
Faculty					
Health sciences	338	67.6	22–53	40.07 ± 6.28	0.03
Others	162	36.4	20–60	38.7 ± 6.44	
Follows a food plan					
Yes	206	41.2	22–52	37.36 ± 6.06	
Ketogenic diet	20	4	22–45	33.25 ± 6.96	0.002
Hypocaloric diet	114	22.8	24–52	36.6 ± 6.12	
Mediterranean diet	24	4.8	25–49	36.08 ± 5.65	
High-protein diet	14	2.8	32–48	41.14 ± 5.33	
High-calorie diet	27	5.4	28–50	38.7 ± 5.45	
Others	7	1.4	29–48	40 ± 6.24	
No	294	58.8	20–60	41.23 ± 6.07	
Has ≥ 2 servings of vegetables/day					
Yes	298	59.6	20–53	39.04 ± 6.28	0.01
No	202	40.4	22–60	40.52 ± 6.38	
Performs physical activity					
Yes	278	55.6	20–53	39 ± 5.99	0.01
No	222	44.4	22–60	40.44 ± 6.71	

BMI: body mass index.

**Table 5:** Multiple regression analyses among ORTO-15 scores and different variables in our population sample.

Model 1	*β* (95% CI)	SE	*p*-Values
Gender [F]	−2.98 (−4.23; −1.73)	0.64	4 × 10^−6^
BMI	−0.41 (−0.57; −0.25)	0.08	6.71 × 10^−7^
Adj.R^2^	0.06		
**Model 2**	***β* (95% CI) **	**SE**	***p*-Values**
Gender [F]	−2.98 (−4.20; −1.76)	0.62	2 × 10^−5^
BMI	−0.27 (−0.42; −0.1)	0.08	0.002
Health science faculty	1.26 (0.15; 2.37)	0.57	0.03
Follows a food plan	−3.14 (−4.29; −1.99)	0.57	1 × 10^−7^
Has ≥ 2 servings of vegetables/day	−0.42 (−1.52; 0.68)	0.56	0.45
Performs physical activity	−1.20 (−2.28; −0.122)	0.55	0.03
Adj.R^2^	0.14		

Adj.R^2^: adjusted coefficient of determination; β: regression coefficient; CI: confidence interval; F: female; SE: standard error.

## Data Availability

The data analyzed in the current study are available from the corresponding author upon reasonable request.

## Supplementary Materials

The following supporting information can be downloaded at: https://www.mdpi.com/article/10.3390/nu17030537/s1, Figure S1: Correlation between ORTO-15 and EHQ-21 in the total sample and categorized by sex.
