# Supplementary material for: Examining Associations Among Orthorexia Nervosa and Anthropometric Factors and Lifestyle Habits in an Italian University Community
Source: Nutrients. 2025 Jan 31;17(3):537. doi: 10.3390/nu17030537 (PMC11819917; doi:10.3390/nu17030537)

Supplementary Figure S1. Correlation between ORTO-15 and EHQ-21 in the total sample or categorized by sex

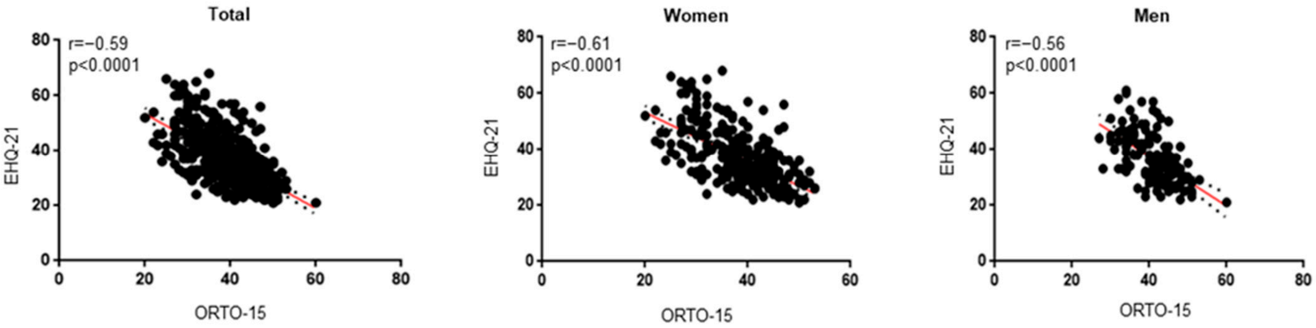

Supplement: Supplementary file 1 [file nutrients-17-00537-s001.zip › nutrients-3428771-supplementary.pdf]
